# Migration and homeostasis of regulatory T cells in rheumatoid arthritis

**DOI:** 10.3389/fimmu.2022.947636

**Published:** 2022-08-09

**Authors:** Konstantin Kotschenreuther, Shuaifeng Yan, David M. Kofler

**Affiliations:** ^1^ Laboratory of Molecular Immunology, Division of Rheumatology and Clinical Immunology, Department I of Internal Medicine, Faculty of Medicine, University Hospital Cologne, University of Cologne, Cologne, Germany; ^2^ Center for Integrated Oncology Aachen Bonn Cologne Duesseldorf, Cologne, Germany

**Keywords:** Treg - regulatory T cells, rheumatoid arthritis, T cell migration, T cell homeostasis, collagen-induced arthritis (CIA)

## Abstract

Regulatory T (T_reg_) cells are garnering increased attention in research related to autoimmune diseases, including rheumatoid arthritis (RA). They play an essential role in the maintenance of immune homeostasis by restricting effector T cell activity. Reduced functions and frequencies of T_reg_ cells contribute to the pathogenesis of RA, a common autoimmune disease which leads to systemic inflammation and erosive joint destruction. T_reg_ cells from patients with RA are characterized by impaired functions and by an altered phenotype. They show increased plasticity towards Th17 cells and a reduced suppressive capacity. Besides the suppressive function of T_reg_ cells, their effectiveness is determined by their ability to migrate into inflamed tissues. In the past years, new mechanisms involved in T_reg_ cell migration have been identified. One example of such a mechanism is the phosphorylation of vasodilator-stimulated phosphoprotein (VASP). Efficient migration of T_reg_ cells requires the presence of VASP. IL-6, a cytokine which is abundantly present in the peripheral blood and in the synovial tissue of RA patients, induces posttranslational modifications of VASP. Recently, it has been shown in mice with collagen-induced arthritis (CIA) that this IL-6 mediated posttranslational modification leads to reduced T_reg_ cell trafficking. Another protein which facilitates T_reg_ cell migration is G-protein-signaling modulator 2 (GPSM2). It modulates G-protein coupled receptor functioning, thereby altering the cellular activity initiated by cell surface receptors in response to extracellular signals. The almost complete lack of GPSM2 in T_reg_ cells from RA patients contributes to their reduced ability to migrate towards inflammatory sites. In this review article, we highlight the newly identified mechanisms of T_reg_ cell migration and review the current knowledge about impaired T_reg_ cell homeostasis in RA.

## Introduction

Rheumatoid Arthritis (RA) is a chronic systemic inflammatory autoimmune disease characterized by a symmetric polyarthritis with subsequent joint destruction and deformity. While typically associated with cartilage destruction and bone erosion of afflicted synovial joints, RA can also present with a myriad of extra-articular manifestations (1). Symptom constellations seen in RA patients can lead to severe disability, with a significant reduction in quality of life as well as employability (2, 3). Considering the overall lifetime risk of developing RA in US-women is 3.6% and 1.7% in US-men (4) as well as an estimated worldwide prevalence of 0.46% (5), the aggregate reduction in quality of life and productivity, as well as increased utilization of healthcare resources have significant societal as well as economic impacts.

With continued research, our understanding of RA and its underlying mechanisms has grown significantly. While the pathogenesis of RA is complex and involves the interplay of many factors, an increasing amount of attention has been given to the role of regulatory T cells (T_regs_), in disease development and progression. As a specialized subset of helper T cells, T_regs_ play an important role in maintaining homeostasis and self-tolerance, thereby preventing the development of autoimmune diseases such as RA. With this review we aim to synthesize the growing body of knowledge available on T_reg_ cell migration and homeostasis as it pertains to RA. An understanding of this topic is not only relevant to the pathogenesis but also potential treatment modalities of RA.

## Rheumatoid arthritis

The multifactorial pathogenesis of RA has been the subject of numerous publications as the exact mechanisms continue to be uncovered. It has been proposed that various environmental factors play a role in the susceptibility to RA, with smoking being identified as the most important environmental risk factor associated with the development of RA others of which include low vitamin D intake and levels as well as occupational dust exposure (6–9). Interestingly, mice with chronic exposure to cigarette smoke showed reduced levels of T_regs_ in peripheral blood *via* flow cytometry (10). By means of genome-wide association studies (GWAS) with subsequent meta-analyses, RA susceptibility has also been linked to major histocompatibility complex (*MHC*) genetic variants with different serological phenotypes of RA being associated with distinct human leukocyte antigen (*HLA*) gene variations. For example, it has been found that the *HLA-DRB1* genotype, associated with increased CXCR4 expression on cluster of differentiation 4 positive (CD4^+^) T cells, a protein which is involved in cellular migration, leads to sustained autoimmunity and local inflammation thereby conferring an increased risk for RA development (11, 12). MicroRNA (miRNA), which is small non-coding RNA involved in the regulation of post-transcriptional gene expression has been proposed as an epigenetic process involved in RA pathogenesis by means of modulating T- and B-cell subtype development and differentiation. While certainly not the only miRNA domain with relevance to RA, it has been shown that microRNAs and their interplay with transcriptional factors can modulate the peripheral blood Th17/T_reg_ balance (13). This is of particular note as RA is heavily associated with a disturbed Th17/T_reg_ balance skewed in favor of the pro-inflammatory Th17-cells.

**Figure 1:**
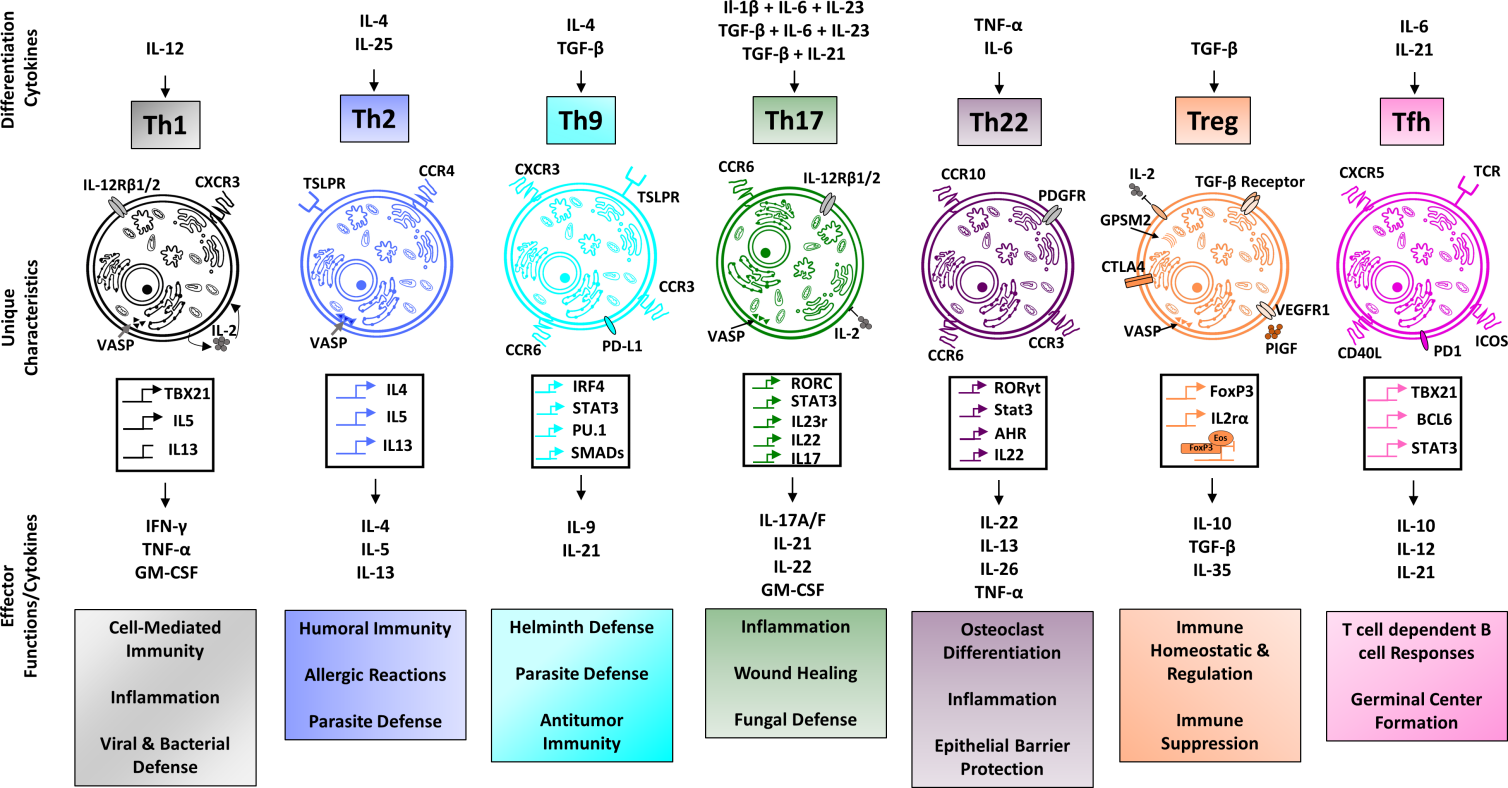
Overview of CD4+ T cell subsets. Regulatory T cells, along with the Th2 phenotype are classically associated with anti-inflammatory functioning, whereas Th1 and Th17 cells are typically associated with pro-inflammatory functions. Th9 cells are implicated in host defense against helminth infections, Th22 cells in host defense against bacterial pathogens and Tfh cells play an important role for the development of lasting immune memory. The individual characteristics and functions of CD4^+^ T cell subsets are made possible through distinct gene profiles resulting in a subtype-specific expression of receptors and cytokine production. Different environmental stimuli, such as TGF-β in the case of regulatory T cells induce the differentiation of naive CD4+ T cells into their respective subtypes (14–17).

It is presumed that the presence of the aforementioned risk factors in combination with a triggering event such as infection or injury is what ultimately causes the development of autoreactive T- and B- cells and subsequent development of RA. While the exact pathomechanisms underlying RA and its associated manifestations are highly complex and multi-factorial, a significant amount of attention has been given to CD4+ T-helper cells (Th-cells), which represent the most abundant lymphocyte population in the synovial infiltrate. Naïve CD4+ T cells can develop into distinct subsets with specific phenotypes and functions (**Figure 1**). Observations have shown abnormalities in intracellular signaling as well as aging of T helper cells in patients with RA, thereby contributing to the chronic autoimmune response associated with RA (18).

RA presents clinically with the bilateral insidious onset of polyarticular symmetric arthritis. Patients experience pain and soft effusion-based swelling in affected joints, primarily the metacarpophalangeal, metatarsophalangeal and proximal interphalangeal joints as well as the wrist. RA, however, is not limited to small joints and can also affect ankle, knee, elbow and shoulder joints. While joint involvement is the classical manifestation associated with RA, inadequate treatment can lead to the development of numerous extra-articular manifestations including, but certainly not limited to cardiovascular disease, interstitial lung disease and liver dysfunction (1). In addition, when considering the mean loss of life expectancy in patients diagnosed with RA is 4.97 years (19) the gravity of the disease becomes clear.

## Regulatory T cells

CD4^+^ regulatory T cells are a distinct T cell subpopulation involved in the maintenance and regulation of self-tolerance and homeostasis. First discovered in 1995, T_regs_ specifically express the transcription factor forkhead Box P3 (FoxP3) in the nucleus as well as CD25 and CTLA-4 on their cell surface (20). Although FoxP3 expression is significant for the differentiation of CD4^+^ T cells to the regulatory T cell phenotype, numerous epigenetic changes have also proven to be crucial for effective T_reg_ functioning. However, FoxP3 holds a pivotal role to regulatory T cell function, as evidenced by the X-Linked syndrome of immune dysregulation, polyendocrinopathy, and enteropathy (IPEX) caused by a mutation in the FoxP3 gene leading to large amounts of polyclonal T-cell activation and tissue infiltration (21, 22). While numerous studies have been able to implicate dysfunctional T_regs_ in autoimmune disease and inflammatory conditions, increasing evidence suggests the role of T_regs_ goes far beyond simply acting as a checkpoint on overarching inflammatory processes. As our understanding of regulatory T cells and their role in disease progresses, increased attention is being given to this cell-type as a potential therapeutic target benefitting disease control. As a key regulator of immune function, both down- and up-regulation of immune responses by means of regulatory T cell modulation could be feasible therapeutic angles for treating numerous diseases.

Although FoxP3 has been shown to be key to T_reg_ differentiation and functioning, further genes play a pivotal role in T_reg_ development and functioning. T_reg_ genome regulators such as SATB1 (Special AT-rich sequence binding protein 1) bind to genomic sites to unwind chromatin and activate super-enhancers. The importance of this genomic organizer to regulatory T cells has been illustrated by experiments showing SATB1-deficient mice not being able to generate T_regs_ in the thymus, as well as the failure of CD25^+^ FoxP3^-^ CD4^+^ T cells deficient in SATB1 to differentiate into FoxP3^+^ T_regs_ under appropriate conditions for differentiation (23). These activated super-enhancers subsequently demethylate and thereby activate the hallmark T_reg_ genes among which are *FOXP3*, *IL2RA* (encoding CD25), *CTLA4*, *IKZF2* (encoding Helios) and *IKZF4* (encoding Eos), collectively known as T_reg_ -specific demethylated regions (T_reg_-DRs). While super-enhancer dependent demethylation is able to upregulate gene expression, FoxP3 expression has been associated with the downregulation of IL2, IFN-γ and Zap70 genes, which interestingly do not possess any of these T_reg_-DRs, thereby suggesting a FoxP3-independent mechanism of T_reg_-DR regulation (24). Both epigenetic changes and FoxP3 expression are regulated in an independent manner by means of TCR activation; however, both are required for a definite differentiation to the T_reg_ lineage.

Although mainly produced in the thymus, T_regs_ also develop in the periphery, such as in the intestinal mucosa. Interestingly, in comparison to other T cell subtypes, T_regs_ leave the thymus in an antigen-primed state, characterized by the expression of CD25, CTLA4 and CD5 (25). In addition to their activated state, upon leaving the thymus, T_regs_ have also been shown to have a high T cell receptor (TCR) affinity for self-antigens (26). By means of their primed state and high TCR affinity for self-antigens, T_regs_ are activated to exert suppressive effects at up to ~100-fold lower peptide/MHC concentrations when compared to other T cell subsets that recognize the same antigen (27). These two characteristics ensure a rapid T_reg_ response towards self-antigens, subsequently preventing other immune cells from being activated and causing an aberrant immunologic response. Once in the periphery, FoxP3^+^ T_regs_ find themselves in a continuous state of high proliferation under physiologic conditions, most likely due to continued recognition of self-antigens as well as antigens derived from ubiquitously present microbes (28, 29). Peripherally located naïve CD4^+^ T cells can differentiate to FoxP3^+^ Tregs under both inflammatory and non-inflammatory conditions following specific antigen exposure (30, 31). While thymic T_regs_ (tT_regs_) are classically associated with the recognition of self-antigens, peripherally induced T_regs_ (pT_regs_) are involved in the recognition of foreign antigens such as bacterial, allergy and food antigens (32). This differentiation is further reflected in the differential expression of Nrp-1, being high in tT_regs_ and low in pT_regs_ as well as in the TCR repertoire, which shows limited overlap among the two subsets (33). Interestingly, it has been shown tT_regs,_ in the absence of pT_regs_, are incapable of suppressing chronic inflammation and autoimmunity (34).

Although classically associated with the suppression of overarching immune-responses, T_regs_ have garnered attention for numerous other functions. While T_reg_ cells play an important role in various autoimmune diseases and contribute to dysregulated immune response in malignant diseases (35–40), they have also been implicated in controlling fetal-maternal tolerance (41–43), immunometabolic disease including obesity and atherosclerosis (44–46), degenerative diseases with inflammatory components as well as tissue regeneration (47). Currently, FoxP3, CD25, and CD45RA are used to identify and divide human circulating FoxP3^+^ CD4^+^ T cells into distinct subsets. Resting or naïve T_regs_ are characterized by CD45RA, as well as low levels of CD25 and FoxP3 expression. Effector T_regs_ are characterized by no expression of CD45RA but high levels of both FoxP3 and CD25 expression. Lastly, CD45RA negative cells with low CD25 and FoxP3 expression are not considered to be T_regs_ (28). As we continue to uncover new functions and mechanisms of T_regs,_ it is becoming clear just how vital this cell type is to maintain physiologic homeostasis in numerous organ systems. With their wide range of functions, T_regs_ continue to be an interesting target for further research both in the understanding, and treatment of RA as well as numerous other conditions.

Multiple mechanisms have been hypothesized by which T_regs_ exert their effector functions, all of which are dependent on FoxP3 expression. It is of note that T_regs_ have been shown to possess a large degree of lineage stability, maintaining their FoxP3 expression and subsequent inhibitory effects under the influence of many different immune stimuli (48). Of note is the ability of T_regs_ to express transcription factors and chemokine receptors typically associated with other T cell lineages, with the subsequent production of IFN-γ (Th-1 like T-regs), IL-17 (Th-17 like Tregs) and IL-13 (Th-2 like T-regs) (49–52). Interestingly, male scurfy strain mice that are hemizygous for the X-linked FoxP3sf mutation, essentially depriving them of T_reg_ functioning, develop a CD4^+^ T cell mediated lymphoproliferative disease characterized by wasting and multi-organ infiltration by lymphocytes (53). This has been highlighted by the work of Fontenot et al. who were able to show that retroviral-driven Foxp3 expression in CD4^+^ CD25^-^ T-cells resulted in protection from disease onset and progression classically associated with the scurfy strain (54). The suppressive effects are postulated to be exerted *via* cell-contact dependent as well as humoral factors including cytokines (IL-2, IL-10, TGF-β), cell surface molecules (CTLA-4, CD25, TIGIT) and intracellular molecules (granzymes, cAMP). Just as numerous as the mechanisms by which they potentially exert their functions are the cells that T_regs_ take effect on, including CD4^+^ and CD8+ T cells, NK cells, B-cells, monocytes and dendritic cells (DCs) (55). Much interest has been given to T_regs_ and their relation to interleukin 2 (IL-2), which has also been referred to as T cell Growth Factor (TCGF). IL-2 together with its receptor (IL-2R) is involved in the maintenance of self-tolerance as well as immunity and has become the target of biologic immune-modulators used in the treatment of autoimmune and rheumatic disease (56). As FoxP3^+^ T_regs_ barely produce any IL-2 due to downregulation of the IL-2 gene by FoxP3 expression, they are dependent on exogenous IL-2 for survival (57). While studies have come to differing conclusions on the matter, a role of T_regs_ as IL-2 “reservoir” has been postulated. Due to T_regs_ having the highest expression level of the IL-2 receptor α-chain among T cells, which is responsible for maintaining the receptor in a high-affinity state T_regs_ can effectively starve other immune cells and APCs of IL-2, thereby inhibiting their activation and preventing an active immune response (58–60). Interestingly, effector T cells produce IL-2 when in the activated state to sustain the immunologic response, which in turn activates regulatory T-cells in order to prevent an overarching immune reaction. In addition to IL-2, other “pro-inflammatory” cytokines such as IL-6 and TNF-α have been shown to induce T_reg_ expansion, indicating the natural role of T_regs_ in controlling immune reactions at sites of inflammation (61). CTLA-4 expression is a further means of T_reg_ functioning, causing the downregulation of both CD80 and CD86 expression in APCs, thereby preventing co-stimulatory signaling of CD28 to activate effector T cells (62). The importance of CTLA-4 mediated effector functioning has been shown by the conditional deletion of CTLA-4 in adult mice, causing spontaneous lymphoproliferation, hypergammaglobulinemia, as well as various autoimmune inflammatory manifestations with accompanying organ-specific antibodies (63).

Although only a selection of T_reg_ functionalities and mechanisms of action have been touched upon in this section, it is evident that T_regs_ can provide an attractive target in the prevention or management of numerous diseases. Due to their ability to significantly alter immune functioning through down-regulation of effector cells, T_regs_ provide us with a powerful means to potentially modulate immune function. For example, the induction of T_regs_ in humans with IL-2 administration to treat disease caused by overreaching immune reactions has shown promising results (64, 65).

### T_regs_ in rheumatoid arthritis

**Figure 2:**
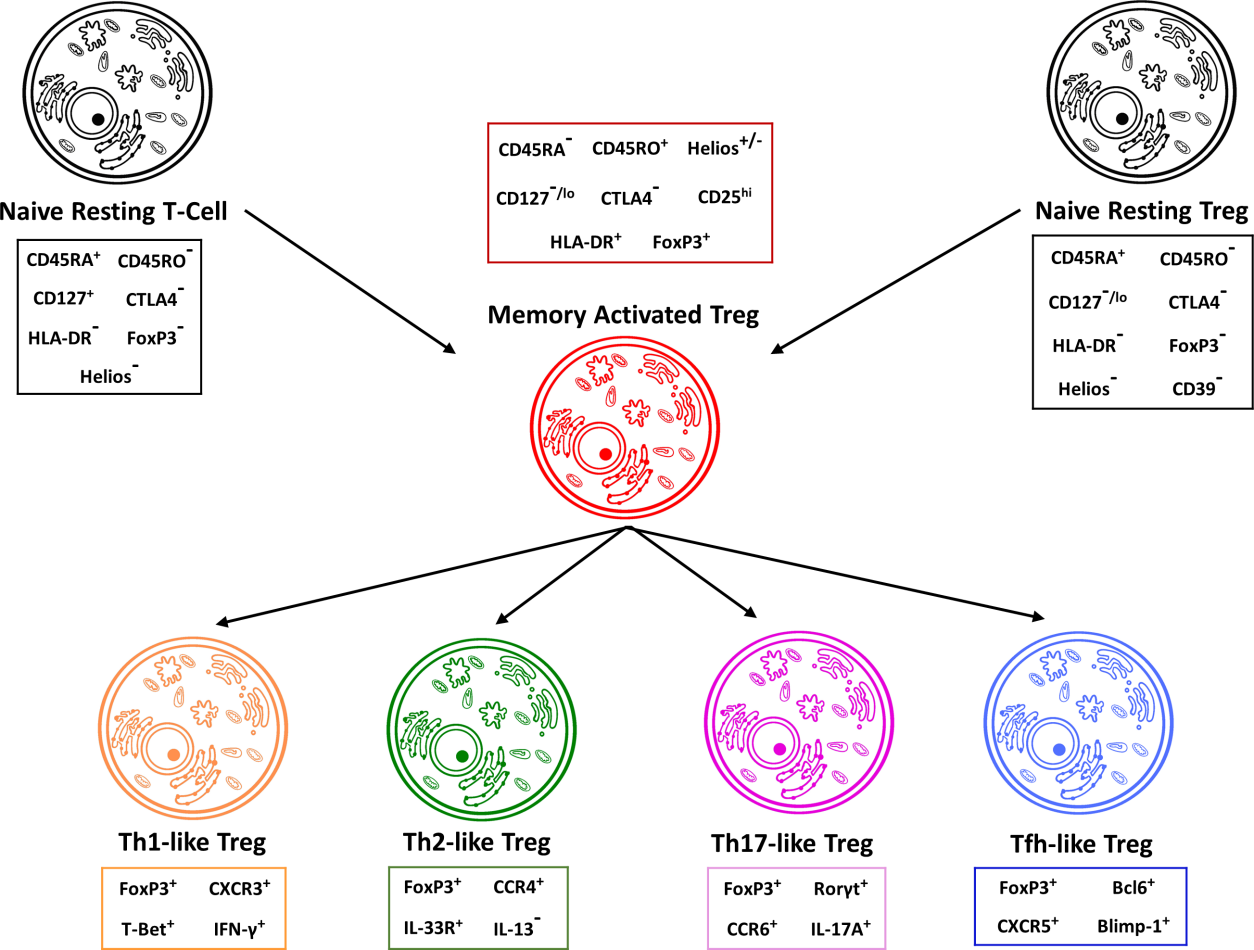
Treg cells can be divided in distinct subgroups. Treg cells can further be subdivided into memory activated, Th1-like, Th2-like, Th17-like and Tfh-like regulatory T cells. Both naïve CD4+ T cells as well as naïve resting regulatory T cells can differentiate into memory activated Tregs, which then further delineate into their specific regulatory T cell phenotypes. These phenotypes can be differentiated through different expression profiles for genes, transcription factors and cytokine production. In addition, the individual regulatory T cell phenotypes have unique immune capabilities and functions. Th17-like Tregs for example are capable of producing IL-17A and have been shown to be over-represented in patients suffering from RA (73, 74).

As we continue to expand on our knowledge in regard to regulatory T cells, they are becoming of ever more interest to the understanding of rheumatoid arthritis. With their immunosuppressive role, regulatory T-cells not only play a key role in the pathogenesis of RA but can also provide valuable insights into possible treatment modalities. Although controversial results have been published, the general consensus is that regulatory T-cell levels are reduced in patients with Rheumatoid Arthritis, which is in line with the general anti-inflammatory effect attributed to T_regs_ (66, 67). While difficult to pinpoint, the difference in experimental results has been attributed primarily to the absence of universal characteristics that define T_regs_. While the most common method of identification for T_regs_ in flow cytometry is CD3+ CD4^+^ CD25^high^/CD127^low^, attempts are being made to identify better mechanisms to reliably and reproducibly identify T_regs_. Helios has been proposed as one of these mechanisms, as its expression in CD4^+^ FoxP3^+^ T cells is negatively correlated to RA disease activity, significantly more so than other cell-surface markers such as CTLA-4. Further work has shown that diverging results may not only result from a lack of homogenous T_reg_ identification, but also on the different expression of specific T_reg_ subpopulations that require individual antibody-staining for flow cytometry. Moreover, some T_reg_ subpopulations show specific features which are characteristics of other CD4+ T-cell subsets. Under certain inflammatory settings, T_reg_ cells can develop into Th1-like, Th2-like, Th17-like, or Tfh-like T_reg_ cells (68–72) (**Figure 2**). Th1-like T_reg_ cells acquire a Th1-like phenotype and express the pro-inflammatory cytokine IFN-γ while they lose their suppressive capacity (49, 75). Similar, Th2-like and Th17-like T_reg_ cells express the transcription factors GATA-3 or RORγt and secrete IL-13 or IL-17 (71, 76). Th2- and Th17-like T_reg_ cells have been reported to maintain their suppressive functions (71, 76). Interestingly, Th17-like T_reg_ cells have been observed in humans under physiological conditions (71). In RA, the frequency of Th1-like Treg cells is increased but deficient in function (75).

The aforementioned Helios as a member of the Ikaros transcription factor family, is a nuclear factor associated with the early development of regulatory T-cells. Studies have shown a significant importance of Helios in controlling the stability and function of T_regs_ (77). Interestingly, most of the FoxP3^+^ CD4^+^ T-cells in the synovium of inflammatory arthritis patients are Helios^+^. In addition, these Helios^+^ T_regs_ prove to be poor producers of effector cytokines including IL-10, IFN-γ and TNF-α. Helios expression seemed to not only affect cytokine production but was also associated with a higher Ki67 index indicating higher levels of cellular proliferation when compared to Helios^-^ T_regs_. The overall expression of FoxP3 gene expression as well as TSDR (regulatory T cell-specific demethylated region) demethylation is decreased in RA individuals when compared to healthy individuals, in contrast to Helios, which shows an overall higher expression in RA patients as compared to healthy individuals. These results suggest that the nuclear transcription factor Helios may act to suppress the inhibitory functions normally carried out by T_regs_ in patients with RA (78). While these results certainly demonstrate the importance of Helios in regards to RA, it is not the only Ikaros zinc finger transcription factor (IkZF) with implications for RA.

Eos, another of the IkZF factors, has garnered attention due to its high expression in T_reg_ populations and function as a vital component of the FoxP3-mediated gene expression complex. Specifically, Eos forms a protein complex with C-terminal Binding Protein and FoxP3 to promote gene silencing, thereby acting as a co-repressor of vital importance to the maintenance of the T_reg_ phenotype as well as its suppressive capabilities. This was further established by means of Eos expression knockdown in mice which resulted in a worsening of inflammation in colitis (79). In addition, the Eos/FoxP3 repression complex has been shown to target and subsequently repress the IL-2 gene locus. Interestingly, Helios has also been attributed with the ability to repress the IL-2 gene locus, signifying redundance in the IkZF Factor family functioning. Just as with Helios, Eos has also been shown to be downregulated as a consequence of increased DNA methylation in T_regs_ of RA patients. This suggests both Helios and Eos play a possible role in the aberrancy of T_reg_ functioning, contributing to the development and progression of RA.

Aiolos, another member of the Ikaros family of transcription factors, is induced in CD4+ T cells by Ahr in the presence of TGF-β1 and has been shown to be upregulated in RA (37, 80). Not only has Aiolos been shown to be upregulated in RA patients, it is also correlated to a higher prevalence of a lymphoid pathotype, as well as higher ACPA and RF prevalence indicating more severe disease. Interestingly, Ikaros and Aiolos synovial expression was directly correlated with synovial cell infiltration as well as systemic inflammation (81). Aiolos levels increase during the β- and positive selection of thymocytes along with Helios and Ikaros. In contrast to Helios and Ikaros, Aiolos levels remained elevated after β- and positive selection throughout the continued thymocyte development (82). Interestingly, it has been reported that treatment with TNF-α inhibitors induces Aiolos in CD4+ T cells (83). With Aiolos being able to transactivate the Bcl-2 promoter, it has the potential to prolong cellular viability by preventing apoptosis. In contrast, IL-2 starvation induces Ras-Aiolos association, thereby inhibiting Aiolos functioning with the subsequent inhibition of BCL-2 expression leading to cellular apoptosis (84). In contrast to Helios and Ikaros which show transient expression in T cells, Aiolos levels remain elevated even as cells continue to mature, which is congruent with its function in preventing apoptosis (80). Interestingly, the expression of Aiolos in T_regs_ that lack Helios expression has been associated with a “pro-inflammatory T_reg_ subtype” that is capable of producing and secreting IFN-γ, IL-2, and IL-17. T_regs_ not expressing Aiolos on the other hand, are better suited for suppression of effector T-cells. Whereas Helios and Eos are co-expressed in T_reg_ cells, Aiolos is expressed in Helios^-^ T_reg_ cells (85).

It has been shown that there is a significant reduction in the proportion of T_regs_ in the peripheral blood of patients with active RA when compared to RA patients in remission, which is an interesting contrast to a higher proportion of T_regs_ in the synovial fluid of RA patients when compared to healthy controls. This might be in part due to the anoxic environment created through synovial inflammatory processes in active RA (86). Interestingly, it has been demonstrated that hypoxia and the subsequent induction of hypoxia-inducible factor 1 alpha (HIF-1α) expression promotes the induction of FoxP3 transcription, which in turn promotes the generation of T_regs_. In addition, CD4^+^ CD25^+^ T cells from inflamed joints express high levels of CTLA-4, GITR, CD69 and MHC class 2 molecules, indicating an activated state. This at first may seem in stark contrast to the observed overarching inflammatory reaction seen in the joints of RA patients. However, it has been shown that RA patients have defective synovial T_reg_ functioning with an inability to suppress not only the production of pro-inflammatory cytokines such as TNF-α and IFN-γ by other CD4^+^ T-cells as well as monocytes, but also a reduced suppression of T effector cell proliferation (87). While only part of a much larger picture, this data can suggest that the dysfunction more so than the absence of T_regs_ plays a role in the pathophysiologic processes dictating RA. We have directly been able to study the effects of regulatory T-cells in RA by proxy of collagen-induced arthritis (CIA) in mice. CD25-depleted mice induced with collagen to promote the development of RA showed significantly more severe disease when compared to control mice. This increased disease severity was underpinned by increased antibody titers against collagen as well as an increased proliferation of collagen-specific T cells. Furthermore, the adoptive transfer of CD4^+^ CD25^+^ T cells into CD25-depleted mice showed an attenuation of disease severity, highlighting the importance that T_regs_ have in controlling aberrant inflammatory responses in joints (88). In addition, the severity of RA symptoms as well as high levels of RF and anti-CCP antibodies has been associated with a lower number of activated T_regs_ in humans (89).

The production of these RF and anti-CCP antibodies occurs through B-cells, which require CXCR5^+^ T follicular helper cells (Tfh) for activation. Interestingly, Tfh cells require activation of the transcription factor Bcl-6, which is upregulated in CD4^+^ T-cells of RA patients, as well as IL-6 and IL-21 for their differentiation (90). The subsequent unregulated activation of antibody producing B cells and associated humoral responses are linked to the development of RA, as well as B cell lymphomas in RA patients (91, 92). In addition, Tfh cells, which are usually located in B cell follicles to regulate B cell survival, are overly abundant in the synovium of RA patients as compared to healthy individuals (93). Expansion of the Tfh population in RA is driven by enhanced IL-6/pSTAT3 signaling and leads to a shift in the ratio between Tfh cells and Tfh-like Treg cells, which share many phenotypic characteristics with Tfh cells, but lack CD40L and IL-21 expression (94). A subset of Tfh cells is derived from natural Treg cells and is characterized by the expression of FoxP3 (95). These cells are referred to as T follicular regulatory cells (Tfr) (95). Blimp-1 dependent (96) ability to suppress the formation of germinal centers as well as limit humoral responses makes Tfr cells an important counter-force to the Tfh-associated RA-promoting actions. Both activated Tfh and Tfr cells migrate to germinal centers, where Tfh cells secrete IL-21 and promote a B cell response through CD40L/CD40 interactions, whereas Tfr cells secrete IL-10 and suppress B cell responses *via* CTLA4 and GITR (97). A deficiency in CXCR5^+^ T_reg_ cells, for example, has been associated with an increase in germinal center B-cells and the associated pro-inflammatory characteristics (95, 98).

## Th17/T_reg_ balance

Although regulatory T cells exert their effect on a multitude of different cell types, the pro-inflammatory CD4^+^ Th17 phenotype, which is characterized by expression of the retinoic acid-related orphan receptor (ROR-γt) and the production of the IL-17 cytokine family is of particular interest when trying to understand the implications of regulatory T cells in RA. The pro-inflammatory Th17 cells, along with pro-inflammatory Th1 cells have been shown to be resistant to CD6 down-regulation in RA, which is associated with an aggravation of autoimmune inflammation as well as increased cell proliferation and survival (99, 100). While long presumed to be a pro-inflammatory Th1-mediated disease, increasing attention is being given to Th17 cells as they pertain to the development and progression of RA. When considering that the inflammatory activity in arthritis has been attributed to Th17 cells in the synovial fluid (101), as well as the number of Th17 cells in the serum of RA patients being positively correlated with the disease activity score in 28 joints (DAS28), anti-CCP antibody and C-reactive protein levels (102), the importance of interaction between T_regs_ and Th17 cells in RA becomes very apparent. Through the production and secretion of numerous cytokines such as IL-17A, IL-17F and IL-22, Th17 cells have been shown to stimulate synovial fibroblasts as well as macrophages to the large-scale production of pro-inflammatory mediators such as IL-1, IL-6, TNF-α and PGE2, thereby worsening synovial inflammation (103). In addition, Th17 cells stimulate synovial stromal and innate lymphoid cells to secrete GM-CSF thereby initiating and elevating joint inflammation (104, 105).

**Figure 3:**
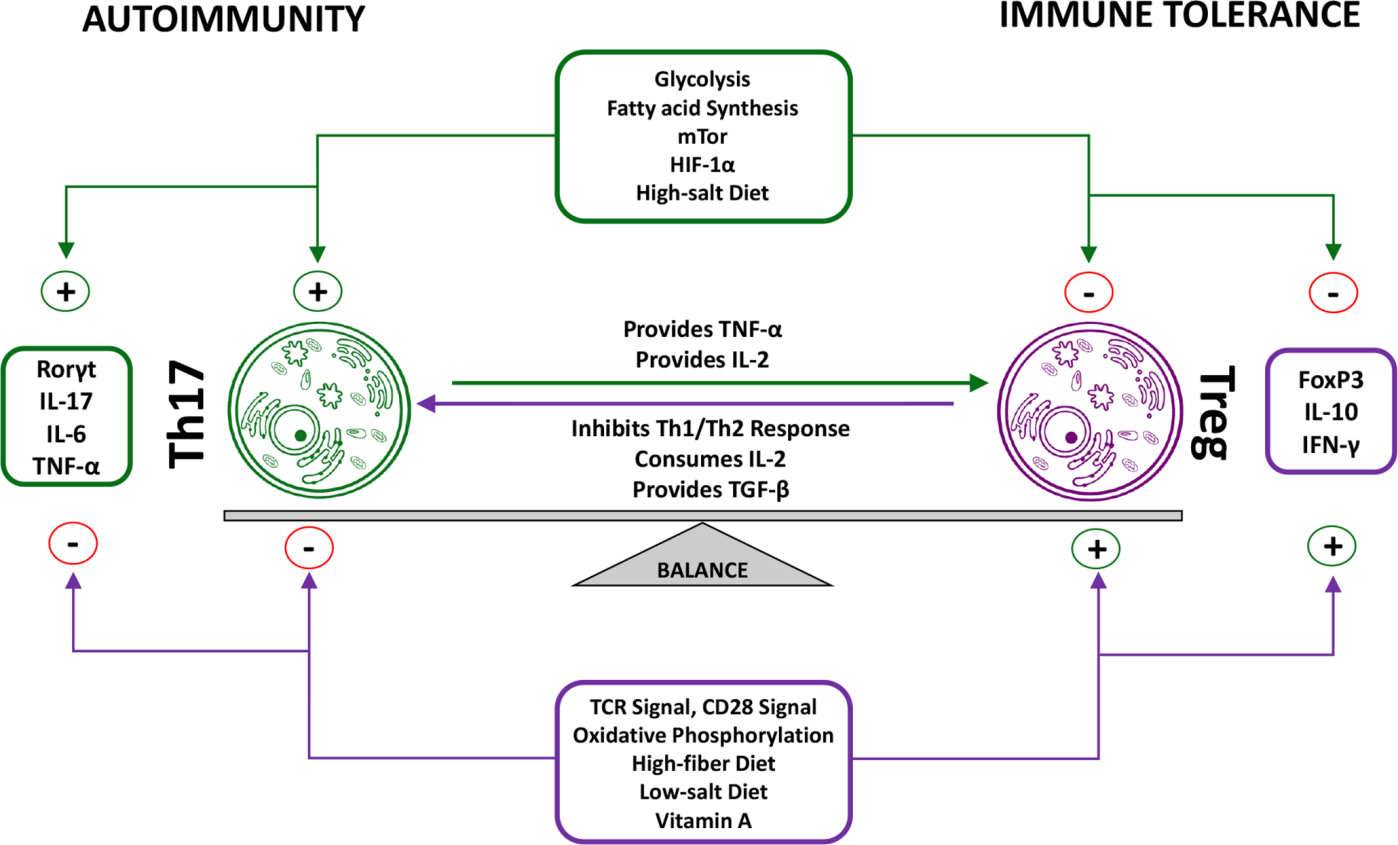
Reciprocal functions of Treg cells and Th17 cells. The balance between Treg cells and Th17 cells is of great importance for proper immune functioning. A dysregulation of this balance in favor of the pro-inflammatory Th17 phenotype has been observed in RA patients. Although various factors influence this balance through isolated effects on either Treg or Th17 cells, numerous factors have been identified that simultaneously take effect on both Tregs and Th17 cells. Furthermore, Tregs and Th17 cells also directly take effect on one another. Th17 functioning physiologically causes an increase in the Treg population, which in turn prevents overarching immune reactions through inhibition of Th17-specific as well as other immune pro-inflammatory functions (15, 16, 74).

While Th17 and T_reg_ cells significantly differ in their functionality, they do share similarities such as the ability of TGF-β to induce their development and differentiation, which is interesting when considering their opposing functionality. While this at first may seem counterintuitive, it can be proposed that this is an innate attempt to prevent overarching inflammatory reactions that could be caused by aberrant-isolated Th17 differentiation (106, 107). The balance between T_reg_ cells and Th17 cells is modified by various factors, including vitamin A, glycolysis, salt concentrations, and cytokines (**Figure 3**). While TGF-β is required for initial differentiation, many other factors play a role in determining the ultimate fate of Th17 and T_reg_ cells. Furthermore, the Insulin-Like Growth Factor 1 Receptor (IGF1R) has been shown to be an important regulator of Th17 *vs*. T_reg_ cell differentiation. IGF1R signaling possess the ability to enhance activation of the AKT-mTOR pathway in order to potentiate Th17 development in addition to suppressing Th17 apoptosis, while simultaneously hindering T_reg_ development. IGF ligands possess the ability to skew Th17 programming towards the expression of pro-inflammatory genes with concurrent inhibition of anti-inflammatory genes; one proposed mechanism by which this is accomplished is an increase in HIF-1α dependent gene expression (108). Interestingly, continued research has been able to show that lifestyle choices, such as a high-salt diet or cannabinoid consumption might be able to skew this ratio in favor of Th17 cells (109, 110). Numerous studies have been able to show that the Th17/T_reg_ ratio is skewed in favor of Th17 cells in RA when compared to healthy controls. Szodoray et al. tracked the Th17/T_reg_ ratio in patients as they progressed from undifferentiated connective tissue disease (UCTD) to a definitive systemic autoimmune disease (SIAD). From healthy controls to UCTD to definitive SIAD the Th17/T_reg_ ratio continually increased, showing a significant increase of the Th17/T_reg_ ratio in the progression from a healthy state to autoimmune disease (111). These findings were in line with results demonstrating a positive correlation between the percentage of Th17 cells and several clinical markers of RA activity such as DAS28, ESR, CRP, anti-CCP RF and ANA. In contrast, a negative correlation between T_reg_ percentages and DAS28, ESR, CRP, anti-CCP, and ANA was observed (112). These observations are congruent with the functions attributed to Th17 and T_reg_ cells respectively, and further demonstrate the importance the Th17/T_reg_ ratio plays in RA patients. However, clinical trials with the monoclonal antibody secukinumab have revealed a limited efficiency of IL-17A inhibition in patients with RA (113). The limitations of secukinumab in RA may be explained by the fact that the expression of IL-17A and its receptors in the synovium of RA patients is very heterogeneous (114). Moreover, secukinumab does not neutralize IL-17F, which is less active than IL-17A when used alone but is as efficient as IL-17A in the presence of TNF-α (115). A combined inhibition of IL-17A and IL-17F might therefore be more effective in RA as compared to IL-17A inhibition alone.

While the Th17/T_reg_ ratio is of interest when considering how regulatory T-cells could inhibit Th17 functioning, thereby reducing inflammatory signaling, there are further points of interest that need to be taken into consideration when it comes to RA. Komatsu et al. were able to demonstrate that FoxP3^+^ T-cells possess the ability to lose their Foxp3 expression (after which they are called exFoxp3 cells) and undergo transdifferentiation into Th17 cells under the influence of IL-6 derived from synovial fibroblasts. These exFoxp3 Th17 cells play a key role in the pathogenesis of autoimmune arthritis and are present in the synovium of patients with active RA. Interestingly, when compared to Th17 cells derived from naïve T cells, exFoxp3 Th17 cells specifically and highly express the transcription factor SOX4, which in turn positively regulates ROR-γt, thereby enhancing lymphoid cell survival (116, 117). In addition to enhanced survival, the high expression of molecules involved in cellular proliferation as well as a high frequency of Ki-67+ cells among the exFoxp3 Th17 population indicate a high proliferative index under arthritic conditions (118). Not only are exFoxP3 Th17 cells longer lived and more active in regards to proliferation, their osteoclastogenic activity is more pronounced when compared to non-exFoxP3 Th17 cells. It has been proposed that the promotion of osteoclastogenesis by exFoxP3 cells is based on an increased expression of RANKL as compared to regular Th17 cells, with exFoxP3 Th17 cells being able to directly induce osteoclastogenesis, independent of fibroblast presence. The ability of exFoxP3 Th17 cells to accumulate and proliferate in inflamed tissue where they stimulate osteoclastogenesis is significant for the progression of RA and an important aspect to understanding the dynamic between Th17 and T_reg_ cells and how they contribute to RA.

The Th17/T_reg_ ratio has become an area of interest when it comes to developing possible therapeutics to treat not only RA, but a wide array of autoimmune diseases that involve a Th17/T_reg_ dysregulation. Certain medications already exist that take influence the Th17/T_reg_ balance such as the IL-6 Receptor Inhibitor Tocilizumab (119), however, there is much therapeutic potential left to be unlocked by attempting to directly target Th17- and T_reg_-related cytokines, cytokine receptors, intracellular signaling pathways, as well as the modulation of T_reg_ and Th17 specific transcription factors. With the exploration of this therapeutic potential, the far-reaching roles of both T_reg_ and Th17 cells beyond RA pathogenesis have to be kept in mind. For example, Th17 cells and their cytokines IL-17 and IL-22 play a crucial role in the maintenance of immune homeostasis at mucosal surfaces, with IL-17 neutralization being accompanied by high rates of serious adverse events and fungal infections (120). Although the latter effect was observed in patients with Crohn’s Disease, it can be hypothesized that these effects would also be seen in other conditions as this effect is not based on the disease in and of itself, but rather therapy mediated dysregulation of Th17 functioning.

## Mechanisms of T cell migration

Much regard is given to T cells and their effector functions in the synovium that ultimately result in the development of active RA. In order to exert their functions however, T cells must migrate to their respective sites of action. While originating primarily in the thymus in younger years, naïve T cell maintenance throughout life is primarily sustained through peripheral T cell division (121). Interestingly, with active thymic output in childhood 10-30% of all CD4^+^ T cells in blood, lymphoid tissue, and mucosal sites are T_regs_ as compared to only about 5% in adults (122). Even decades after thymic output ceases, naïve T cells comprise a significant proportion (20-50%) of total T cells in multiple lymph nodes. Interestingly, post neonatal thymectomy adults do not have increased incidences of autoimmunity or allergy when compared to age matched controls and are able to maintain T_reg_ frequencies, indicating that while it does hold an important role in T cell development, the thymus is only part of the bigger T cell picture (123, 124). Regardless of age, T_regs_ in blood as well as lymphoid tissues are CD45RA^+^CCR7^+^, indicating a naïve phenotype as compared to mucosal T_regs_, which are CD45RA^-^ and more closely resemble conventional memory T cells (125).

The infiltration of the synovium by CD4^+^ T cells, but not CD8+ T cells or B-cells is necessary for the development of clinically active RA (126). This is of note as a profound understanding of the mechanisms leading to synovial infiltration can be of value in researching potential therapeutic modalities aiming to prevent the onset of RA, rather than combating symptoms of inflammation in manifest disease. Increasing evidence suggests that while not the priming site for naïve T cells, CD4^+^ T cell commitment occurs at the inflamed joints under the influence of cytokines produced by an uncharacteristically large number of activated macrophages and dendritic cells (127). Dendritic cells from RA patients secrete higher levels of inflammatory chemokines such as CCL17, which is involved in the recruitment of CCR4^+^ cells to the inflamed joint (128). Furthermore, monocyte derived dendritic cells from RA patients have been shown to possess an increased capability of Th17 differentiation induction as compared to healthy controls while simultaneously lacking the ability to efficiently induce FoxP3^+^ T_reg_ cells (129). Interestingly, the inability to form succinate from succinyl-CoA due to SUCLG2 repression characteristic of RA in the tricarboxylic acid (TCA-) Cycle with subsequent accumulation of Acetyl-CoA has been shown to skew naïve CD4^+^ T cells to the short-lived effector subtype (SLECs). These SLECs are characterized not only by their hypermobility and ability to rapidly enter synovial tissue with induction of aggressive synovitis, but also by their propensity to proliferate (130).

**Figure 4:**
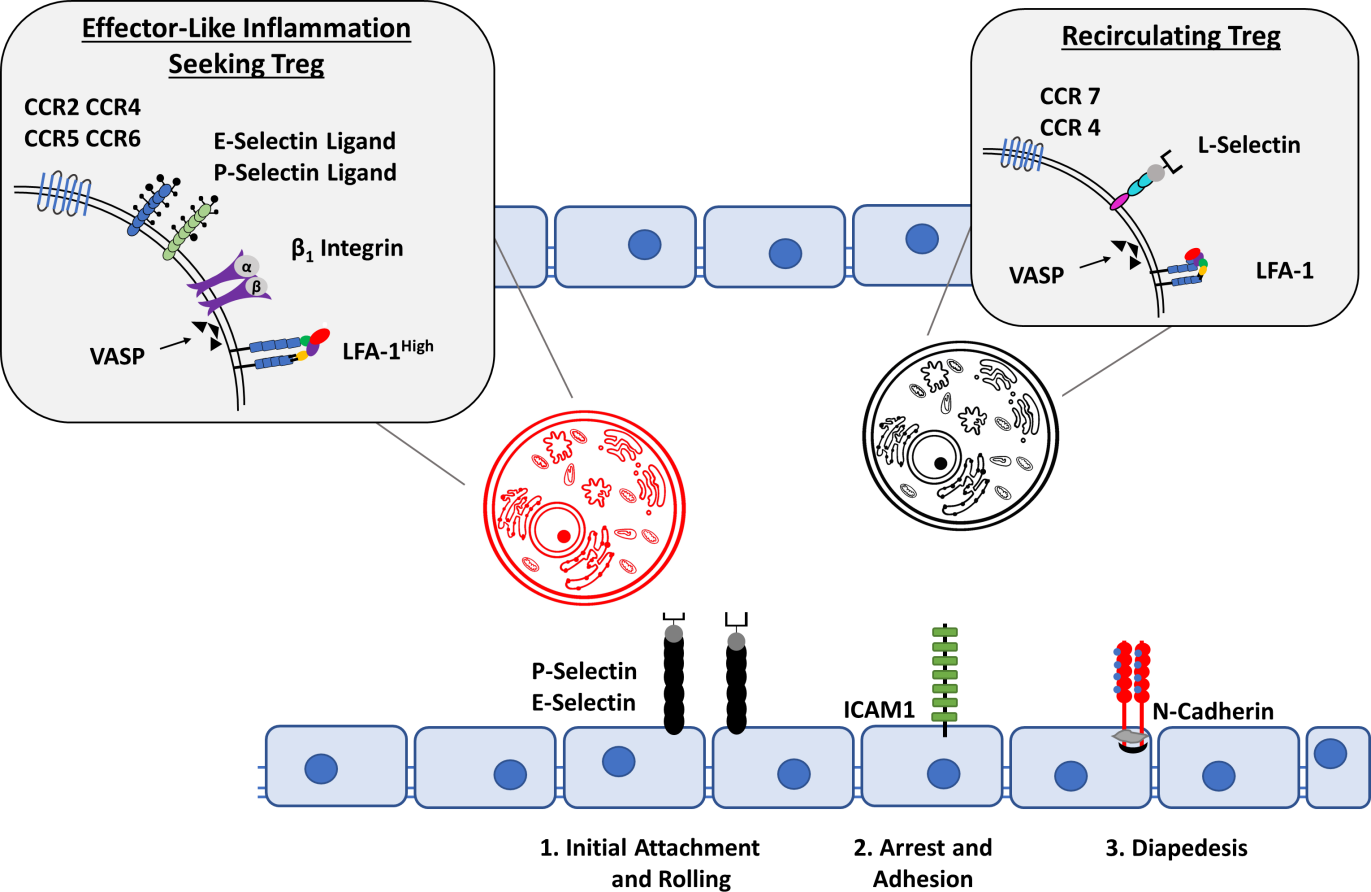
Treg cell migration into inflammatory sites. A dysregulation of Treg migration has been implicated in the pathogenesis of RA. Depending on the expression of cell-surface molecules, Tregs can be divided into recirculating Tregs and inflammation seeking Tregs. In contrast to the expression of CCR7, CCR4, L-Selectin and LFA-1 in a low-affinity state allows recirculating Tregs to remain in the bloodstream, activated inflammation seeking Tregs are able to migrate to their sites of action through the expression of E- and P- selectin Ligands, CCR2, CCR4, CCR5, CCR6, β_1_ Integrin as well as LFA-1 in a high affinity state. The Interaction with P- and E- selectin, ICAM1 and N-Cadherin among other molecules on the activated endothelium allows for the classical steps of cellular migration: Initial attachment and rolling, followed by arrest and adhesion which eventually leads to cellular diapedesis (14, 133).

For T cells to exert their effector functions at sites of inflammation, T cell trafficking consisting of rolling, adhesion and transmigration must occur. For this to take place, cells must express adhesion molecules and chemo-attractans such as selectins (L-selectin for leukocyte populations) as well as the P-selectin glycoprotein ligand-1 (PSGL-1) (131). L-selectin and PSGL-1 interaction allows for leukocyte-leukocyte interactions permitting the tethering and adhesion of leukocyte clusters to the endothelium *via* endothelial specific selectins (P- and E-selectins) of blood vessels prior to extravasation. In addition, lymphocyte function-associated antigen 1 (LFA-1) is of importance for Treg migration, being expressed in a high affinity state in inflammation seeking T_regs_ when compared to recirculating T_regs_ (132). Of note is not only the increased expression of E-selectins at inflamed synovial sites, but also the increased serum levels of P- and L-selectins in RA patients **(** **Figure 4** **)** (134, 135). This suggests an increased ability of leukocytes to migrate to the synovium in patients with RA as compared to healthy individuals. After initial leukocyte-endothelial interaction *via* selectins, integrins are responsible for the firm adhesion to, and arrest of leukocytes at the endothelium. Leukocyte integrins are able to interact with endothelial surface ICAM-1 or VCAM-1, which is a prerequisite for subsequent cellular extravasation. Different immune cell subtypes express individual repertoires of integrins, which are further upregulated in the presence of proinflammatory cytokines such as IL-1 or TNF-α (136). This individualized expression permits the specific localization of T cell subtypes in the inflamed joint. While essential to the extravasation and subsequent navigation to inflamed synovial sites, interactions between leukocyte integrins and their ligands also induce cellular proliferation, cytokine production, and angiogenesis, thereby significantly contributing to disease development (137). The use of specific antagonists to integrins as well as their ligands, has been able to prevent inflammation and angiogenesis in the CIA mouse model; furthermore high levels of soluble and endothelium-bound ICAM-1 have been identified in RA patients, as well as being linked to disease activity in CIA model mice (138–140). Finally, transendothelial chemotactic concentration gradients of molecules such as PECAM-1, ICAM-2, JAMA and ESAM to name a few, allow trans- and paracellular leukocyte migration beyond the endothelium into inflamed tissues (141). This process is regulated by the differential and specific expression of cell trafficking molecules unique to individual cellular subsets, for example, LFA-1 expression, which plays a key role in the emigration of T cells from the vasculature, is upregulated in effector T cells when compared to naïve T cells.

While the migration of T cells in general is of continued interest in research pertaining to RA considering the potential therapeutic value, increased attention is also being given specifically to T_reg_ migration mechanisms, which are diverse and dependent on the cells developmental stage, role, and target tissue. T_regs_ express numerous receptors for inflammatory chemokines as well as adhesion molecules which are not only vital for access to sites of inflammation, but also under heavy regulation. As migration is an energy-intensive process, T_regs_ are reliant on glucokinase-mediated glycolysis, which is supported by the inhibition of motility by means of glucose starvation, and the upregulation of the insulin receptor in supporting their increased energy consumption (142). Glycolytic feedback control through PI3K-Akt pathways has also been demonstrated with the activation of Akt causing a downregulation of L-selectin, CCR7 and sphingosine-1-phosphate receptor 1, implying that the activation of Akt could cause the failure of leukocyte homing to secondary lymphatic organs, instead promoting migration to peripheral tissue such as the synovium (143). Glycolysis interference through extracellular sodium-lactate and lactic acid has also been shown to trap CD4^+^ and CD8^+^ T cells at sites of infection through repression of their mobility (144). In contrast, inhibition of fatty acid oxidation had no impact on T_reg_ migration (133). The PDK-1 signaling-dependent loss of phosphatase and tensin homolog (PTEN) can also take effect on T_reg_ migration through decreasing the expression of CD62L as well as CCR7 (145). The Tumor Necrosis Factor Receptor 2 (TNFR2) has been shown to promote a remarkable degree of cellular migration, activation and proliferation through downstream initiation of the reciprocal PI3K/Akt pathway as well as canonical and non-canonical NF-kB activation (146). The serine threonine kinase mTOR has been identified as a critical component for regulatory T cell migration as well as stability. A specific deletion of mTOR in mouse T_regs_ led to the spontaneous development of severe and systemic inflammation, impaired T_reg_ migration, as well as a loss of FoxP3 expression. Compared with controls, mTOR-deficient T_regs_ expressed increased levels of CD62L, CCR7 and S1P1, as well as decreased levels of CD69, which inhibits the migration from lymphoid tissue to the blood. Furthermore, mTor-deficient T_regs_ expressed lower levels of CD44 and CCR4 as well as slightly lower levels of CCR6 and CXCR3, possibly impairing access to non-lymphoid tissues. This suggests that a deficiency or downregulation of mTOR can cause a defect in entry to nonlymphoid tissues and promotes the recirculation to and within lymphoid organs (147).

## Altered chemokine and cytokine milieu in the synovium

Given their central role in the specific recruitment and activation of T cells as well as other immune cells, chemokines have gained attention as potential treatment targets in RA. As a subgroup of the cytokine family, chemokines are chemotactic and immune modulatory molecules secreted in order to control not only the positioning of, but also the development, homeostasis and activity of numerous immune-competent cells. Interestingly, varying chemokine production profiles have been identified throughout different RA disease stages. CCL4, CXCL4, CXCL7 and CXCL13 expression are characteristic of early RA, whereas CCL3 and CCL9 were indicative of later disease stages (148, 149). Chemokines present in the synovial fluid of RA patients are characterized by high levels of citrullination, especially CXCL5 and CCL2, which causes an altered activity profile with subsequent increased recruitment of monocytes. In addition, the citrullination of CXCL5 has been shown to have a high correlation with RA disease activity (150).

Chemokines have been shown to play a central role in Th1 cell migration to the synovium, which is of note when considering the importance of Th1 cells to the inflammatory processes in RA (151). In addition to Th1 cells, chemokines such as CCL20, which are strongly expressed in the inflamed joint, allow Th17 migration to the synovium with subsequent activation. T_regs_ enjoy a widespread distribution throughout lymphoid and non-lymphoid tissues which is vital to their immunosuppressive function. This propensity to be distributed throughout the body can be altered by means of CCR4 chemokine receptor disruption with the subsequent development of pathogenic inflammatory responses (152). Interestingly, the relational expression of certain chemokines, such as CCR6 with other chemokines has been observed to correlate with the specific production of cytokines. CCR4^+^/CCR6^+^ Th cells express high IL-17A levels whereas CXCR3^+^/CCR6^+^ cells show low IL-17A expression, but elevated IFN-γ levels. CCR6^+^/CCR10^+^ Th cells express high levels of IL-22, thus indicating a connection to the Th22 phenotype, which is characterized by its IL-22 expression. This, however, does not seem to be a uni-directional relationship with IL-17 as well as IFN-γ having been shown to upregulate the expression of numerous chemokines. Interestingly, it has been shown that the presence of a dinucleotide polymorphism in the CCR6 gene is linked to RA susceptibility (153). However, further cytokines such as IL-1β and TNF-α have also been shown to induce chemokine expression, especially CXCL8 and CCL13, both of which act as chemoattractants for various immune cells including T cells and neutrophils (154).

CCL2, also known as monocyte chemotactic protein 1 (MCP1) is produced by synovial chondrocytes and fibroblasts and possesses the ability to recruit CCR2^+^ T cells, NK cells, basophils and macrophages to the synovium. While shown to be upregulated in the synovium of RA patients, the mechanisms underlying CCL2 functioning in RA are complex. This is supported by varying outcomes of CCL2 targeting studies where monoclonal antibodies specific to CCL2 were able to reduce ankle swelling in CIA as well as MRL-lpr mice. In contrast, CCL2 monoclonal antibody treatment aggravated RA during the progression phase in a murine CIA mouse model (155–157). CCL3 and CCL5 are two of the chemokines whose expression is induced by the activation of T cells *via* IL-1β and TNF-α (158). While the receptor for CCL3 and CCL5, CCR5, is not commonly expressed in PBMCs of RA patients, it has been found to be highly expressed in the RA synovium, either due to an upregulation of expression, increase in CCR5^+^ cells in the synovium, or an interplay of both (159). The importance of CCR5 becomes apparent when considering a CCR5 antagonist initiated days before the clinical arthritis manifestation was able to attenuate leukocyte migration to joints thereby reducing disease severity (160). Furthermore, increased levels of CCL3, CCL4 and CXCL10 can be identified in the synovial fluid and plasma of RA patients, whereas CCL5 only seems to be elevated in the plasma but reduced in contrast in the synovium of RA patients (67, 161–164). CXCR3, which is involved in the migration of Th1 cells through interaction with its ligands CXCL9, CXCL10 and CXCL11, has also been shown to correlate with IFN-γ production in its expression, and can reduce susceptibility to inflammatory autoimmune disease when knocked out in mice (165, 166). Furthermore, M1 macrophages have been shown to secrete CXCL5, CXCL8, CXCL9, CXCL10 and CXCL13 which further promotes leukocyte recruitment thereby aggravating joint destruction in RA (167, 168). CCR7 and its ligand CCL21 have also been implicated in the migration of not only T cells but also APCs to the synovium, thereby contributing to RA pathogenesis. CCL21 is of further interest in the regard that it is able to drive osteoclastogenesis in RA through M1 macrophage polarization of Th17 cells as well as the induction of neovascularization (169). CCL20, on the other hand, is secreted by chondrocytes, synoviocytes and Th17 cells in the joints, working in synergy with RANKL to promote the resorption and destruction of bone, as well as contributing to the chemotaxis towards joints of T cells, monocytes, and CD1α^+^ dendritic cells (170–173). CXCL12 was also shown to induce osteoclastogenesis by means of TNF-α-dependent RANKL upregulation in CD4^+^ T cells and synovial fibroblasts. CXCL12 and its receptor CXCR4, are correlated with the presence of CD4^+^ T cells in the synovium, indicating an additional role in T cell migration and joint destruction.

RA is characterized by dysregulated cytokine production and the serum concentration of several cytokines is altered in RA (**Table 1**). As one of the main pro-inflammatory cytokines, TNF-α plays a central role in RA. While the production of TNF-α is classically attributed to macrophages, numerous cells are capable of producing TNF-α. The TNF-α receptor is also widely expressed, including on immune cells. RA is characterized by increased levels of TNF-α in the serum of patients, which carries a central role in the development of the observed inflammation. TNF-α is of great importance due to its ability to stimulate the proliferation and differentiation of T cells, B-cells and NK-cells as well as inducing the production of other pro-inflammatory cytokines such as IL-1, IL-6, IL-8 and matrix metaloproteinases (MMPs). Furthermore, it upregulates the production of GM-CSF, prostaglandins, and collagenases as well as ICAM-1 by synovial fibroblasts (199–202) The importance of TNF-α for regulatory T cells in RA is highlighted by studies showing an increased T_reg_ suppressive activity as well as an increase in T_reg_/T_eff_ balance under treatment with the TNF-α antagonist Infliximab as well as a reduction in T_reg_ numbers in TTG mice overexpressing hTNF-α. TNFR2^+^ T_regs_ isolated from human peripheral blood have been found to exhibit a more potent suppressive effect than TNFR2^-^ T_regs_, further suggesting an interplay between TNF-α and T_regs_ with relevance to RA pathogenesis. The expression of TNFR2 on T_regs_ increased with the progression of disease, suggesting a possible shedding function by T_regs_ to neutralize unbound TNF-α (203, 204).

**Table 1:** Abnormal cytokine expression in RA.

CYTOKINE	FUNCTION(S)	DYSREGULATION IN RA
**TGF-β**	Induction of T_regs_ (174, 175) Induction of SMAD7 (176)	Elevated in RA (177)
**CCL3**	Ligand for CCR5 (178) Involved in regulatory T cell recruitment (179)	Elevated in RA (180)
**CCL4**	Ligand for CCR5 (181) Chemoattractant for T_regs_ (182)	risk allele for RA (183) Upregulated in RA (184)
**IL-2**	Promotes expansion of T_regs_ (185) involved in Treg suppressive function (59)	Increased serum levels in RA (186)
**IL-9**	Enhances T_reg_ cell function (187) Recruits mast cells (188)	Serum and synovial IL-9 levels are increased in RA (189) IL-9 facilitates osteoclastogenesis in RA (190)
**IL-10**	Induces STAT3 activation in T_regs_ (191) Involved in protecting tissues from T cell mediated autoimmune disease Promotes T_reg_ differentiation (192)	IL-10 is elevated in RA patients High serum IL-10 levels are associated with RF and anti-CCP positive RA (193)
**IL-35**	Involved in the induction of a potent Treg cell population (194) directly suppresses effector T cells (195)	High serum and synovial fluid levels correlate with low disease activity in RA (196, 197) reduced in RA (198)

Numerous cytokines and chemokines have been implicated in the development and progression of RA, including cytokines and chemokines that take specific effect on regulatory T cells. Through dysregulation of TGF-β, CCL3, CCL4 IL-2, IL-9, IL-10 and IL-35, connections can be drawn to the functioning, or rather dysfunctioning of Tregs, with the associated negative consequences for patients and their outcomes. As our understanding of cytokine dysregulation, specifically with a focus on Tregs, in RA continues to increase, so does our capability of identifying potential treatment targets. Although only one axis of a multi-faceted disease, influencing Treg functioning capability can yield anti-inflammatory effects to combat the inflammation associated with RA.

Transforming growth factor-Beta (TGF-β) which is expressed by regulatory T cells, is critical in maintaining self-tolerance and immune homeostasis through its involvement in the regulation of cellular proliferation, differentiation, migration and survival (205). TGF-β is able to induce the expression of FoxP3, thereby promoting the differentiation of CD4^+^ T cells to the T_reg_ phenotype (206). In addition, T_regs_ are the primary producers of TGF-β1, as well as being the only cell capable of activating this cytokine *via* expression of cell surface docking receptor glycoprotein A repetitions predominant (GARP) and α-V integrins (207). TGF-β is produced in an inactivated pre-pro-TGF-β precursor form which requires additional stimuli for the liberation and activation of TGF-β prior to exertion of its functions either through cell-surface binding to a heterodimeric receptor complex consisting of type 1 and type 2 trans-membrane serine/threonine kinase subunits, or acting in a soluble form (208). Intracellular signal transduction is subsequently accomplished by SMAD proteins, however SMAD-independent mechanisms of signal transduction have also been identified (209). Subsequently, TGF-β possesses the ability to suppress T cell proliferation *via* inhibition of IL-2 production. In addition, TGF-β modulates the T cell proliferation by alteration of cell cycle regulator expression, specifically causing an upregulation of the cyclin-dependent kinase inhibitors p15, p21 and p27, as well as downregulation of cell cycle-promoting factors such as CDK2, cyclin E and D2, as well as c-myc (210–215). TGF-β is further able to inhibit effector cytokine production by pro-inflammatory Th1 cells through various mechanisms, without affecting the anti-inflammatory effector cytokine production characteristic of the Th2 T cell phenotype (216). TGF-β also affects the differentiation of naïve T cells to different T-helper subsets, further potentiating its immune-modulatory effect (217). In addition to its effects on CD4^+^ T cells, TGF-β also controls CD8+ T cell effector functioning and proliferation by means of inhibiting the production of effector molecules such as IFN-γ and granzyme (218–221).

Interleukin-4 (IL-4) is an interleukin classically associated with anti-inflammatory effects which promotes the differentiation of naïve T cells to the Th2 phenotype, a phenotype which in turn further produces IL-4. With studies having come to different conclusions on IL-4 in RA, some suggesting no-to-minute expression in synovial fluid and other studies suggesting a relevant expression, it remains to be seen what role IL-4 plays in joint inflammation (222, 223). The need for additional studies is highlighted by the differential effects of IL-4 administration in RA models, with IL-4 administration being able to attenuate proteoglycan induced arthritis by means of inhibiting pro-inflammatory cytokine production, although having no disease modulating effect in collagen induced arthritis mice. While the aforementioned results paint a varied picture, IL-4 gene polymorphisms have been found to increase the risk of developing RA in European as well as Chinese individuals making them suitable for the use as a genetic marker to assess the susceptibility to, as well as the subsequent severity of RA (224–226). The role of IL-4 in the homeostasis of T_regs_ is clearer, with IL-4 playing an important role for the suppressive function of T_regs_. IL-4 knockout mice showed a lower expression of granzyme B, which is important for T_reg_-mediated immune regulation, as well as promoting T_reg_ survival, which has been shown through increased cell death percentages in T_regs_ treated with anti-IL-4 antibodies during activation (227).

High levels of IL-7 have been found in the synovial fluid of RA patients when compared to osteoarthritis patients. IL-7, after binding the IL-7R, causes phosphorylation events that cause downstream effects by means of altering Janus kinases (JAK1, JAK3) and STATs (STAT-5a, STAT-5b). JAKs are a group of intracellular non-receptor tyrosine kinases involved in cytokine-mediated signaling *via* the JAK-STAT pathway. While inducing the expression of numerous cytokines, including Il-1a, IL-1b, IL-6, IL-8, TNF-α and macrophage inflammatory protein in monocytes, IL-7 has also been shown to increase the responsiveness of CD4^+^ T cells, thereby lowering the ability of regulatory T cells to suppress them (228).

Although classically associated with Th9 cells, IL-9 is also produced in high quantities by activated T_regs_. The produced IL-9 is essential for mast cell recruitment to tolerant tissue and subsequent mast cell functioning, making IL-9 the functional link between T_regs_ and mast cells (229, 230). In addition, IL-9 is able to prolong neutrophil survival, increase MMP-9 production, and promote the differentiation of Th17 cells, thus cementing its role in the pathogenesis of RA (231). These findings are consistent with the work of Ciccia et al. showing that an increased expression of IL-9 and IL-9R is found in synovial tissue of RA patients as well as correlating with the severity of tissue inflammation (232). Interestingly IL-9 has also been credited with playing a role in the resolution of chronic inflammation in an RA mouse model (233). It has been hypothesized that IL-9 may play different roles in the setting of inflammation depending on its localization and origin. With the expression of CCR3 and CCR6 responsible for trafficking to inflammatory sites, Th9 cells are considered inflammation promoting in autoimmune settings, which lies in stark contrast to T_regs,_ which are classically associated with the attenuation of an inflammatory state (234).

Human cytokine synthesis inhibitory factor (CSIF), more commonly known as IL-10 functions to inhibit the formation of pro-inflammatory cytokines such as TNF-α, IL-1α, IL-1β, IL-6, IL-8, IL-12 and GM-CSF, as well as reducing the expression of HLA-DR and B7 molecules subsequently inhibiting macrophage antigen presentation in the synovial fluid and peripheral blood of RA patients, which in turn attenuates inflammation (235). Furthermore, IL-10 is able to suppress the production of IL-2, thereby causing a broad inhibition of the T cell immune axis which has been reflected in studies showing an attenuation of disease in CIA mice after IL-10 injections (236). In accordance with this, SNPs associated with lower IL-10 mRNA expression are overrepresented in patients with RA (237). IL-10 also holds importance for the differentiation of T_regs_, with studies showing that IL-10-producing B-cells possess the ability to induce T_reg_ differentiation from naïve CD4^+^ T cells (238). In addition to being induced by IL-10, regulatory T cells also possess the ability to produce and secrete IL-10.

The IL-17 cytokine family, most prominently represented by IL-17A, which is produced by Th17 cells, promotes the production of pro-inflammatory cytokines such as GM-CSF, IL-6 and IL-8 from fibroblasts as well as epithelial and endothelial cells. Furthermore, IL-17 is able to promote neutrophil recruitment leading to the aggravation of inflammatory responses (239). First discovered in synovial fluid in 1999, various *in vivo* studies of RA models as well as human *in vitro* studies were able to support IL-17s critical role in promoting inflammation in RA, with newer studies linking IL-17 gene polymorphisms to early RA onset (240). Furthermore, IL-17 was able to increase IL-6, IL-8, CCL2, CXCL1, VEGF and MMP-1 production by RA synoviocytes. It has been found that upon activation, regulatory T cells are able to induce differentiation of CD4^+^CD25^-^ naïve T cells as well as other T_reg_ cells to the Th17 phenotype in the presence of IL-6, thereby promoting the production of IL-17 and subsequently promoting inflammation. This effect was observed independently of TGF-β presence, suggesting that the environment can determine T_reg_ fate and plays a significant role in determining a regulatory T cells role in either attenuating inflammation, or promoting it *via* increased IL-17 production (241). Although numerous studies have been able to establish the importance of Th17 cells and their associated IL-17 in RA thereby making it a potential therapeutic target of interest, IL-17 inhibition has not delivered the hoped-for results. While IL-17A blockade has been shown to be an effective RA treatment when compared to a placebo with ACR20 responses as indicators of efficacy, the effects were only modest (242). Furthermore, when combining TNF with IL-17 inhibition, no significant change in treatment outcome was observed as compared to anti-TNF (adalimumab) treatment alone (243). These results need to be considered when deciding on how much importance should be attributed to IL-17 as a potential therapeutic target in RA.

Interleukin-35, considered to be part of the IL-12 family, is an inhibitory cytokine produced by regulatory T cells (244). Serum IL-35 levels as well as mRNA expression have been shown to be lower in RA patients as compared to healthy controls, with serum levels being negatively correlated to the erythrocyte sedimentation rate and DAS28 of RA patients. In contrast, a positive correlation was found between serum IL-35 levels and T_reg_ frequencies, which seems logical considering T_regs_ themselves are capable of producing IL-35. Osteoclast formation and bone loss induced by TNF-α, as well as the production of the pro-inflammatory cytokines IL-17A and IFN-γ have been shown to be inhibited by IL-35 (245). This suggests that IL-35 enjoys a protective role in regards to RA development, and that its dysregulation is involved in the pathogenesis and clinical manifestation of RA (246).

## IL-6 induced post-translational modification of VASP

Interleukin-6, which has both pro- and anti-inflammatory properties, is produced by numerous cells including T- and B-cells. Associated with the IL-6 specific receptor (IL-6Ra) as well as gp130, a signal transducer, downstream signaling of IL-6 interaction in target cells occurs *via* JAKs. While classical transmembrane receptor associated signaling is considered to carry anti-inflammatory effects, IL-6 trans signaling associated with the soluble form of the IL-6R is given pro-inflammatory characteristics (247). This in turn has given rise to therapeutics that target JAKs in order to attenuate pro-inflammatory effects conveyed by an overabundance of this cytokine. While certainly effective in the treatment of RA, we were able to show that in comparison to conventional biological DMARDs and MTX, which showed the ability to restore T_reg_ numbers in the peripheral blood of RA patients, JAK inhibitors had no effect on T_reg_ levels. This suggests that the T_reg_ number is only one of many factors contributing to RA as a disease (119). High levels of IL-6 have been identified in both blood and synovial fluid of RA patients contributing to inflammation through inducing neutrophils to secrete reactive oxygen intermediates and proteolytic enzymes, as well as inducing osteoclast differentiation *via* a RANKL-independent mechanism. As with IL-4, multiple IL-6 gene polymorphisms have been linked to increased susceptibility as well as clinically more aggressive RA (248). Interestingly, IL-6 has been shown to repress T_reg_ differentiation while simultaneously inducing the development of Th17 cells, thereby skewing the T_reg_/Th17 ratio towards a more pro-inflammatory state (249). Furthermore, the IL-6R is upregulated on Th17 cells in contrast to other CD4^+^ T cells from RA patients, especially so in untreated RA patients, suggesting a role of IL-6 in the retention of transcriptional as well as functional identity of Th17 cells.

Through its ability to reduce the phosphorylation levels of vasodilator-stimulated phosphoprotein (VASP), an important regulator of T cell migration, IL-6 is able to interfere with T_reg_ migration to sites of inflammation, thereby preventing the exertion of T_reg_ suppressive effects at target sites. mIL-6R mediated IL-6 signaling regulates the expression level of VASP that is phosphorylated at Ser157. Recently, Yan et al. were able to show in a mouse model a negative correlation between relative p-VASP expression and IL-6R expression in a CIA transgenic human IL-6 mouse model. It has been shown in HMEC-1 cells, that VASP levels were reduced following *in-vitro* cultivation in the presence of IL-6, thereby establishing a link between T cell migration and IL-6 influence (250). Interestingly, this interference was unique to regulatory T cells, with CD4^+^ effector T cell migration not being affected. This effect was only observed in RA patients, not in healthy controls (251). VASP, as part of the Ena/VASP family, is a cytoskeletal effector protein involved in the coordination of monomeric actin recruitment to the barbed end of the actin filament, thereby preventing actin filament capping as well as playing a role in linear actin polymerization and filament bundling, giving it a vital role to cellular migration (252, 253). Several phosphorylation sites have been identified in the three proteins that comprise the Ena/VASP family, which are regulated by kinases such as PKA, PKG, and PKD1 (254–256). The N-terminal EVH1 domain of VASP regulates cellular localization, whereas the C-terminal EVH2 is involved in tetramerization by binding F-actin thereby facilitating actin polymerization, which in turn is dependent on differential phosphorylation states (257, 258). Phosphorylation levels of VASP were significantly reduced in CIA mice as compared to healthy controls, and subsequently restored through IL-6 receptor blockade (251). These observations were also made in PBMCs of RA patients, presenting with an upregulation of IL-6 as well as a decreased phosphorylation of VASP in untreated RA patients as compared to RA patients treated with IL-6 receptor blockers (251). IL-6 receptor blocker treatment, in addition, was able to increase the frequency of T_regs_ in the peripheral blood of RA patients (251).

Proteomic analysis was able to demonstrate the effects of altered VASP phosphorylation levels with enrichment of proteins involved in integrin signaling, L1 signal transduction, MAP2K and MAPK activation, mitochondrial protein import, protein localization as well as integrin cell surface interactions. Of specific note is the differential protein expression with regard to integrin signaling, whereby patients with low p-VASP expression showed an upregulation in these proteins when compared to IL-6 receptor blocker-treated RA patients with high p-VASP expression. This suggests not only the importance of integrin signaling in the pathogenesis of RA, but also possible therapeutic approaches in regard to altering cellular migration *via* means of IL-6 receptor blockade (251). VASP phosphorylation also has effects on the genomic level, with low p-VASP expression being implicated in the alteration of pathways involved in integrin mediated signaling, integrin binding, leukocyte migration, cell-substrate adhesion, cell matrix adhesion, positive regulation of epithelial cell migration, regulation of substrate adhesion-dependent cell spreading, as well as the regulation of tissue remodeling. These results go to show that the phosphorylation levels of VASP, which are modulated by different IL-6/IL-6R levels, carry importance for both the proteomic as well as genomic homeostasis in RA patients.

While required for T cell diapedesis and trafficking, normal T cell development and the trafficking of naïve T cell populations to the lymph node and spleen occurs independent of VASP. Deletion of VASP in T cells results in an impairment of the alpha 4 integrin (CD49d) with subsequent impairment of activated T cell diapedesis, suggesting the effects of VASP on transendothelial migration being CD49d-dependent. Although diapedesis is inhibited through impaired VASP functioning, T cell adhesion to, as well as crawling on endothelium is not affected (259). First strides are being made in investigating the therapeutic potential of VASP inhibition in the prevention of cancer metastasis development. VASP might also prove to be an interesting target in the treatment of RA by means of promoting regulatory t cell migration to sites of inflammation, as well as potentially inhibiting the migration of pro-inflammatory acting effector T cells (260).

## Lack of GPSM2

G-protein-signaling modulator 2 (GPSM2) also known as LGN, is involved in the modulation of G-protein-coupled receptor functioning, thereby altering the cellular response initiated by cell-surface receptors in response to extracellular signals. The N-terminal half contains 10 copies of leu-gly-asn amino acid repeats, hence the alternative LGN naming, as well as four GoLoco motifs on the C-terminal end which are involved in guanine nucleotide exchange (260). GPSM2 is classically associated with the regulation of cell division and the cell cycle as well as the development of normal hearing (261). GPSM2 mutations have been shown to cause non-syndromic hearing loss and deafness as well as contributing to mechanisms underlying common brain malformations, as well as finding implications in the cellular migration of malignant cells, the latter of which indicates a role of GPSM2 in aberrant cellular migration (262, 263).

Recently, the understanding of GPSM2 functioning was expanded by observations implicating GPSM2 in the pathogenesis of RA *via* means of altered T_reg_ migration. Meyer et al. were able to show that phosphorylation of serine/threonine kinases in CD4^+^ T cells is significantly altered as compared to healthy controls. The phosphorylation level of GPSM2 is reduced in CD4^+^ T cells from RA patients and is significantly downregulated in experimental autoimmune arthritis following immunization of mice with collagen type II (264). Interestingly, treatment with anti-IL-6 receptor antibodies restores the phosphorylation level of GPSM2 in CD4^+^ T cells from RA patients (264). The changed phosphorylation level after treatment with anti-IL-6 receptor antibodies could be related to reduced IL-6 signaling or could be caused by a general reduction of inflammation (264). Phosphorylation of GPSM2 was shown to be significantly downregulated in untreated RA patients as compared to healthy controls and RA patients treated with IL-6 receptor inhibitors, making it a potential therapeutic target in the treatment of RA. This notion is further supported by the finding that GPSM2 expression is not only downregulated, but completely lost in CD4^+^ T cells four weeks following the immunization of mice with collagen type 2 in the induction of experimental autoimmune arthritis. While it was shown that loss of GPSM2 expression correlated with increased paw size in CIA mice, there was no correlation to radiographic signs of bone erosion (264).

The vital role of GPSM2 in cellular migration, and specifically T_reg_ migration was established through the ability to significantly inhibit T_reg_ migration through GPSM2-specific antibodies in healthy individuals. Interestingly, blockade of GPSM2 in patients with active untreated RA had no effect on T_reg_ migration. This lack of effect in untreated RA patients can possibly be attributed to the lack of GPSM2 expression in the setting of untreated RA as established in the CIA mouse models. In contrast, T_regs_ from RA patients treated with IL-6 receptor inhibitors showed increased T_reg_ migration when compared with untreated RA patients and healthy controls, as well as the ability to alter T_reg_ migration *via* antibody-mediated GPSM2 inhibition. While implicated in regulatory T cell migration, specific blockade of GPSM2 showed no significant influence on overall CD4^+^ T cell migration. This data suggests an interplay between IL-6 receptor signaling, GPSM2 expression and subsequent inhibition of cellular migration, which can be attenuated by means of long-term IL-6 receptor inhibition. These findings have been able to implicate GPSM2 as a promoter of cellular migration specifically in T_regs_, without taking effect on other CD4^+^ T cell subsets. While T_reg_ migration is significantly reduced through a blockade of GPSM2, it is not completely eliminated, suggesting a certain amount of redundancy within the regulation of T_reg_ migration that can compensate for a loss of GPSM2. GPSM2 thereby is another axis of the IL-6 receptor effects implicated in regulatory T cell homeostasis and migration, along with, among others, modulation of the NF-kB signaling pathway (264).

## Discussion

Regulatory T cells have been able to attract increased attention in research related to autoimmune diseases, to which rheumatoid arthritis is no exception. T_regs_, through their immune inhibitory properties, prove to be of great importance when it comes to understanding the pathogenesis and discovering possible treatment targets for RA. As our population continues to age and the prevalence of RA continues to increase in the western world, the significance of advancing our understanding of and treatment strategies for RA becomes more apparent. With this systematic literature review, we provide an overview of the latest understanding of the homeostasis and migration of T_reg_ cells in RA.

As a CD4^+^ T cell subset with anti-inflammatory properties and immune inhibitory effects, T_reg_ cells provide an important counterpart to overarching inflammatory processes. Dysregulation or inability to carry out the innate functions of T_regs_, therefore, can lay the foundation for the development of diseases in the autoimmune spectrum such as RA. Regulatory T cells have been shown to play a vital role as an anti-inflammatory axis in the physiologic as well as diseased state, the modulation of which could prove effective in both prevention and treatment of RA. By means of affecting both environmental cytokine profiles and the functioning of numerous immune-competent cell types, T_regs_ can be considered a central player in the aberrant processes leading to the development and progression of not only RA, but various other diseases.

Numerous chemokines and cytokines, both affecting regulatory T cells, have been implicated in the development and progression of RA. These include both pro-inflammatory cytokines such as IL-6, IL-17, IL-1 and TNF-α, as well as anti-inflammatory cytokines such as IL-4 and TGF-β, to name a few. Chemokines such as CXCL5 and CCL2, among others, with relevance to regulatory T cells, have also been implicated in RA through their increased citrullination in the synovium, with CXCL5 citrullination levels enjoying a close correlation to disease activity. While taking direct effect on pro- and anti-inflammatory effector functions, altered chemokine and cytokine levels also affect regulatory T cell migration to sites of inflammation. RA is characterized by an altered ability of regulatory T cells to migrate to sites of inflammation, which in part can be explained by a lack of GPSM2 and VASP activity due to post-translational modifications and altered phosphorylation states.

Many of these already serve as therapeutic targets through monoclonal antibodies targeting TNF-α (Infliximab, Adalimumab, Golimumab), the IL-6 receptor (Tocilizumab) and the IL-1 receptor (Anakinra). As our understanding of RA and regulatory T cells continues to grow, so too does the list of potential targets for the therapeutics of tomorrow. Furthermore, this growing understanding might one day be able to help us prevent RA onset before a need for therapeutics arises.

## Author contributions

KK, YS, and DK performed literature research and prepared the manuscript. KK prepared the figures. KK and DK wrote the manuscript. All authors contributed to the article and approved the submitted version.

## Conflict of interest

The authors declare that the research was conducted in the absence of any commercial or financial relationships that could be construed as a potential conflict of interest.

## Publisher’s note

All claims expressed in this article are solely those of the authors and do not necessarily represent those of their affiliated organizations, or those of the publisher, the editors and the reviewers. Any product that may be evaluated in this article, or claim that may be made by its manufacturer, is not guaranteed or endorsed by the publisher.
